# Profile of children with COVID-19 infection: a cross sectional study from North-East Nigeria

**DOI:** 10.11604/pamj.supp.2020.35.145.25350

**Published:** 2020-08-13

**Authors:** Idris Abiodun Adedeji, Yusuf Misau Abdu, Mohammed Faruk Bashir, Adamu Saidu Adamu, Garba Dayyabu Gwarzo, Bashar Salisu Yaro, Auwalu Abdullahi Musa, Zuwaira Ibrahim Hassan, Aliyu Mohammed Maigoro, Yusuf Bara Jibrin

**Affiliations:** 1 Department of Paediatrics, Abubakar Tafawa Balewa University Teaching Hospital, Bauchi, Nigeria,; 2 Department of Community Medicine, Abubakar Tafawa Balewa University, Bauchi, Nigeria,; 3 Department of Paediatrics, Bayero University, Kano, Nigeria,; 4 Ministry of Health, Bauchi, Nigeria,; 5 Infectious Diseases Unit, Department of Internal Medicine, Abubakar Tafawa Balewa University Teaching Hospital, Bauchi, Nigeria

**Keywords:** COVID-19, children, sub-Saharan Africa, Nigeria

## Abstract

**Introduction:**

available evidence suggests that children infected with COVID-19 tend to have a less severe form of the disease. However, most of the studies that have established this largely emanate from outside sub-Saharan Africa. The pandemic nature of the infection makes it instructive to evaluate its pattern among children across different climes, including ours. This study was set out to describe the clinical characteristics of children with COVID-19 in Bauchi State, North-East Nigeria.

**Methods:**

this was a cross sectional study that involved 53 children between the ages of 0 and 18 years, who had RT-PCR confirmed COVID-19 infection between March and June 2020 in Bauchi State, Nigeria. Data on epidemiological and clinical characteristics was analysed using IBM SPSS Statistics V 21.® Relationship between categorical variables was established using the chi square test. The level of statistical significance was set at < 0.05, at a confidence interval (CI) of 95%.

**Results:**

the mean age was 12.63 ± 4.31 years with a slight preponderance of males (1.1: 1). Majority were asymptomatic (60.4%), while 32.1% and 7.5% had mild and moderate diseases respectively. The most common symptoms were cough (20.8%), fever (17%), and sneezing (15.1%). Five children (9.4%) complained of loss of taste while anosmia was documented in one child (1.9%). We observed a significant relationship between age category and the presence of symptoms. In fact, children younger than 10 years (pre-adolescents) were five times more likely to be symptomatic when compared to those above this age (p = 0.029, C I 1.08-21.56).

**Conclusion:**

our findings have shown a mild pattern of disease and good outcome among infected children. However, we must be mindful of the higher vulnerability among younger children, especially those below 10 years.

## Introduction

The Coronaviruses (CoV) are a large family of single-stranded enveloped RNA viruses that belong to the family of *Coronaviridae* and order *Nidovirales* [1]. They are zoonotic and may cause serious respiratory, cardiovascular and gastrointestinal diseases as well as coagulopathy, multi-organ failure and death when humans are infected [2]. In 2002, there was the emergence of the novel CoV severe acute respiratory syndrome (SARS CoV), while in 2012 the world witnessed an outbreak of the Middle East respiratory syndrome coronavirus (MERS-CoV) [3,4]. The 2019 novel Coronavirus (COVID-19) which originated from Wuhan City, Hubei Province in China is currently responsible for a global pandemic which by the end of May 2020, had affected over 190 countries and territories, and had infected over six million people, out of which about four hundred thousand have had fatal outcomes [5]. Within the same period, Nigeria had recorded over 10,000 cases of COVID-19 with a mortality rate of about 3% [6]. As at the end of June 2020, Bauchi State, North East Nigeria, had about 500 recorded cases of COVID-19, a significant percentage of these were children below the age of 18 years [6]. In spite of the rapidly developing body of knowledge on the characteristics as well as the therapeutics of this condition, there is still a dearth of information on how children are affected by COVID-19, especially in sub-Saharan Africa. Available facts from outside Nigeria suggest that fewer children are affected by the virus with many of them being asymptomatic and with rare records of mortalities, as compared to the adult population [7,8]. The differences have been postulated to be the result of changes within both immune function and the angiotensin-converting enzyme (ACE) 2 receptor, used by the virus to enter type II pneumocytes in the lung, as a result of aging. Decreases in ACE-2 seen in animal models of aging have been shown to result in changes in neutrophil influx and resultant lung injury [9]. Thus, immunosenescence and changes in inflammatory responses with age likely account for the different spectrum and severity of disease in children.

In a report from China, 2.4% of those infected were children and adolescents below 19 years, while according to a survey carried out by the United State Centre for Disease Control (US-CDC), 1.7% of 149,760 laboratory confirmed cases were observed among children and adolescents below the age of 18 years [8,10]. The median age of infection from a study in the US was 7 years, furthermore, a slight male preponderance has been observed among infected children in the US and in China [10,11]. In spite of the evidence that have shown that most children are spared from the severe manifestations of this condition, they nonetheless play a vital role in the community spread of the virus, as it has been observed that more children are likely to develop upper respiratory tract infections with nasopharyngeal carriage [12]. The aim of this study was to evaluate the clinical profile of children and adolescents between the ages of 0 and 18 years, who had laboratory confirmed COVID-19 in Bauchi State between March and June 2020. Beyond understanding the characteristics of COVID-19 among children in this region, the findings from this study may influence the decisions that would guide the control of COVID-19 especially among the paediatric population of Nigeria and by extension, sub-Saharan Africa.

## Methods

This cross sectional study was conducted in Bauchi State, North-East Nigeria, among 53 children between 0 and 18 years, who had RT-PCR confirmed COVID-19 diagnosis. Currently, the state adopts targeted community contact testing strategy, where mass testing is done in communities observed to have a high rate of community transmission. Majority of the children diagnosed in Bauchi State were identified through contact tracing, however, 25% presented primarily at health facilities. Thirty-six of the 89 children were excluded because of incomplete data. All the children recruited were diagnosed and managed according to the Nigeria Centre for Disease Control (NCDC) COVID-19 protocol, [13] between March and June 2020. Ethical approval for this study was obtained from the Bauchi State Health Research Ethics Committee (protocol approval number: NREC/03/11/19B/2020/32). Informed consent was obtained from the parents and assent was gotten from the older children. The following were applied in categorizing disease severity among the infected children [14]; 1) Asymptomatic infection; Children tested positive for 2019-nCoV, but without manifestations of clinical symptoms or abnormal chest imaging findings. 2) Mild disease/Acute upper respiratory tract infection; Children with only fever, cough, pharyngeal pain, nasal congestion, fatigue, headache, myalgia or discomfort, etc., and without signs of pneumonia by chest imaging or sepsis. 3) Moderate disease/Mild Pneumonia; Children with or without fever, respiratory symptoms such as cough; and chest imaging indicating pneumonia, but not reaching the criteria of severe pneumonia. 4) Severe disease; Meeting any of the following criteria. a) Increased respiratory rate: ≥ 70 times/min (< 1 year), ≥50 times/min (≥1 year) (after ruling out the effects of fever and crying); b) Oxygen saturation <92%; c) Hypoxia: assisted breathing (moans, nasal flaring), cyanosis, intermittent apnea; d) Disturbance of consciousness: somnolence, coma, or convulsion; e) Food refusal or feeding difficulty, with signs of dehydration. 5) Critical cases; Those who meet any of the following criteria and require ICU care: a) Respiratory failure requiring mechanical ventilation; b) Shock; c) Combined with other organs failure. Information on epidemiological and clinical profile of the enrollees was obtained with the aid of a semi structured interviewer administered questionnaire. All data generated were processed and analysed using IBM SPSS Statistics V 21®. The chi-square test and odds-ratio were used to establish the association between categorical variables. The level of statistical significance was < 0.05, at a confidence interval (CI) of 95%

## Results

**Figure 1:**
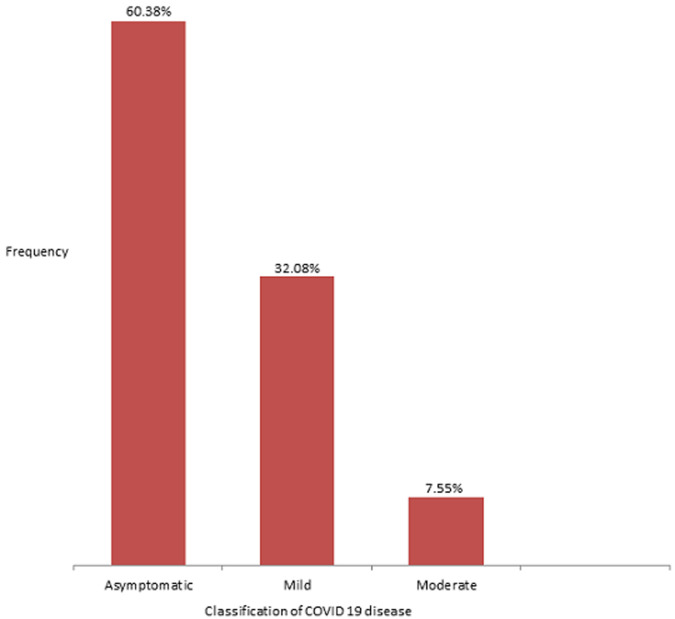
bar chart showing the classification of the severity of Paediatric COVID-19 disease

**Figure 2:**
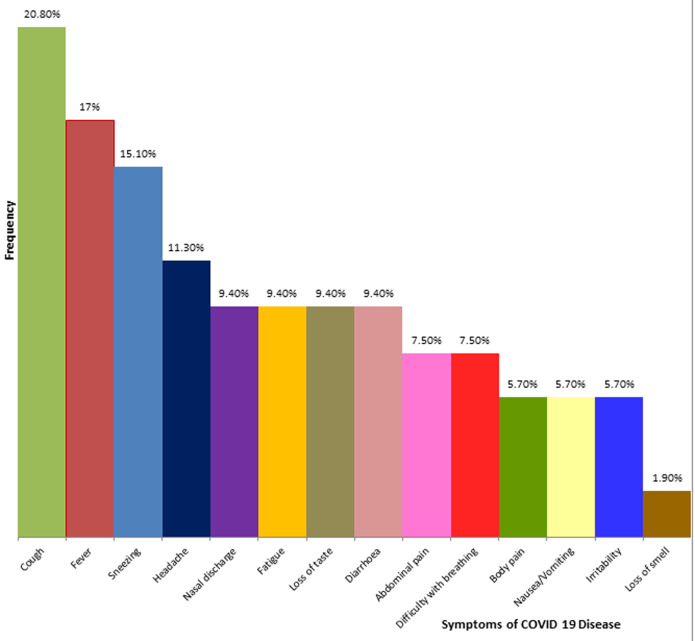
clustered bar-chart showing the frequency of symptoms

There were a total of 495 confirmed cases of COVID-19 infection from March to the end of June 2020 in Bauchi State. Out of these, 89 were children between the ages of 0 and 18 years, which represented 18% of the total population. However, 36 children were not eligible, as they did not have complete data. Thus, this study analysed the clinical profile of 53 cases of paediatric COVID-19 infection. The subjects had a mean age of 12.63 ± 4.31 years, with their ages ranging from 1.5 to 18 years. There is a slight preponderance of males (1.1: 1) and 81.1% of the enrollees were adolescents (Table 1). Most of the cases were asymptomatic (60.4%). Furthermore, all the symptomatic children had either a mild or moderate disease, as there was no case of severe or critical disease (Figure 1). In addition, the most commonly reported symptoms were cough, fever and sneezing (Figure 2). A higher percentage of the infected pre-adolescents were symptomatic and there is a significant relationship between the age group of the infected children and the presence of symptoms. Indeed, the odds of an infected pre-adolescent being symptomatic are five times greater than same occurring among adolescents (Table 2).

**Table 1:** age and sex distribution of subjects

			Sex		Total
			Male	Female	
**Age category (years)**	Pre-adolescents	0-4	1(50)	1(50)	2(100)
		5-9	5(62.5)	3(37.5)	8(100)
	Adolescents	10-14	8(36.4)	14(63.6)	22(100)
		15-19	14(66.7)	17(33.3)	21(100)
**Total**			28(52.8)	25(47.2)	53(100)

Mean age: 12.63 ± 4.31 years

**Table 2:** relationship between the age category of children with COVID-19 and the presence of symptoms

		Presence of symptoms			X2		Odds Ratio	
		Symptomatic	Asymptomatic	Total	Value	p	Value	CI
**Age category**	Pre-adolescents	7 (70.0%)	3 (30.0%	10 (100.0%)	4.754	0.029	4.83	1.08-21.56
	Adolescents	14 (32.6%)	29 (67.4%	43 (100.0%)				
	Total	21 (39.6%)	32 (60.4%)	53 (100.0%)				

## Discussion

In this study, we have described the demographic and clinical characteristics of 53 children who were diagnosed and subsequently managed for COVID-19 disease in Bauchi, Nigeria, between March and June 2020. Interestingly, they accounted for 10.7% of all the cases recorded during this period. This is considerably higher than what has been documented in previous reports across different regions, since the emergence of the virus in Wuhan. According to the Chinese Centre for Disease Control and Prevention, which reviewed the data of 72,314 confirmed cases, only 1.3% was accounted for by children below 19 years [15]. Similarly, the USA Centre for Disease Control reported that of the 149,082 cases confirmed by April 2020, 1.7% was among children below 19 years [8]. However, these are countrywide surveys that involved a significantly larger number. Furthermore, it is quite possible that the apparently higher infection rate among the paediatric age group from our study is as a result of a testing pattern, especially during contact tracing, which may have been skewed towards children. The fertility rate in Bauchi is one of the highest in the country, with many homes housing a reasonably large number of children below the age of 18 years [16]. Thus, it is quite likely that the proportion of tested children as compared to adults in many clusters would be higher than what obtains in other climes. Moreover, the paucity of regional and nationwide data on paediatric COVID-19 limits a comparative evaluation of the epidemiological pattern, and the appreciation of the true rate of infection among the children in our setting.

Our study has found that majority of the infected children were adolescents, with a mean age of 12.63 ± 4.31 years. This appears to be at variance with the findings from a multinational study of paediatric COVID-19 in Europe [17], where the median age was 5 years, as well as an earlier report from Wuhan [18], which identified a median age of 6.7 years. A preliminary analysis from the USA had however documented a median age of 11 years [8], which is comparable to our finding. Although the explanation for this varying trend is not clear, it may be attributable to factors such as the sample size and scale of the different surveys, as well as the sources of infection among the children. Indeed, it would be instructive to assess the impact of the source of infection on the age at risk of contracting the virus among children, especially in our setting. The protective influence of parents on younger children and a less stringent adherence to the myriad of protective measures among the youths may also be contributory factors. The slightly higher ratio of infected males in our study is similar to the findings from previous surveys [15,17]. It has been established that males are at increased risk of more severe disease and fatal outcome [19]. Angiotensin converting enzyme (ACE) receptors are central to the pathogenesis of COVID-19 as they are required for the entry of the viruses into the cells [1]. Studies have shown that the expression of these receptors is higher in adult males. Furthermore, oestrogen has been proven to have a negative influence on the activities of the receptors [20]. These may perhaps explain the protection that females tend to exhibit against the virus. Our study was however not able to establish any significant relationship between gender difference and neither the presence of symptoms, nor severity of disease.

The children in our study were mostly asymptomatic (60.4%), while the rest had mild (32.1%) or moderate disease (7.5%). A larger study in China which involved 728 infected children reported 90% as having asymptomatic, mild or moderate disease [10]. The most common symptoms in our study were cough (20.8%), fever (17%) and sneezing (15%). Anosmia was documented in one child while five children had gastrointestinal symptoms. Earlier evidence has suggested that children are largely spared from the severe forms of COVID-19 disease, with a significantly higher number of asymptomatic infections relative to the experience among adults [15]. This has been attributed to differences in immunological response to the infection. Cross-immunity from previous exposure to viruses, including other corona viruses, reduced occurrence of up-regulation of ACE 2 receptors from co-morbid or pre-morbid diseases, as well as a better alveolar regenerative capabilities and lower viral load, are some of the factors that may explain a milder pattern of disease in children [21]. Contrary to our observation, an earlier survey from Europe had documented that only 19% of the children they studied were asymptomatic [17]. However, this survey was conducted among children who were treated within the hospitals and who according to the authors, represented the severe end of the disease spectrum. It is also worthy of note that a significant proportion of the children in the European based survey had long standing underlying illnesses (25%) and co-infection by other viruses (5%). Such was not the case in our study, as most of the children that had the infection were otherwise healthy and were only detected through contact tracing. Furthermore, similar to our findings, the most commonly reported symptoms from the aforementioned studies were cough and fever [8,10,17].

A higher proportion of the infected children whose ages were less than 10 years were symptomatic. In fact, these pre-adolescent children were five times more likely to manifest symptoms when compared with the adolescents. Gotzinger *et al*. [17] had earlier observed that among infected European children, those who were more likely to develop a severe disease and subsequently required admission into the Intensive Care Unit (ICU), were the children younger than 10 years (68%). The explanation for these differences within the paediatric age groups is not yet fully understood. However, it is hoped that there will be more clarity as new bodies of information on this topic continue to emerge.

There were limitations encountered in the course of carrying out this study. We were only able to access complete data of 53 out of the 89 children that were confirmed to have the COVID-19 infection during this period. This represented only 60% of the positive children. Furthermore, some of the information we obtained was retrospective and thus subject to recall bias. Lastly, obtaining information about the presence of certain symptoms from children below the age of five can be understandably challenging. Hence, it is possible that some of the symptoms in our study were under-reported.

## Conclusion

Our study has shown that children of all ages in our environment may be at a high risk of contracting COVID-19, although, the vast majority are likely to present with mild symptoms or remain asymptomatic. Care must however be exercised, particularly with respect to the younger children, who have been shown to be significantly at risk of increased vulnerability.

### What is known about this topic

- COVID-19 is a pandemic infection that also affects children;
- Most children that are infected present with a mild form of the disease with very low fatality rates.

### What this study adds

- The findings from this study reveal that COVID-19 infection in children is mostly a mild disease;
- When pre-adolescents are infected, they have much higher odds of having a symptomatic course, on comparison to adolescents.
